# Cognitive impairment and edentulism among older adults: an observational study using claims data

**DOI:** 10.1186/s12877-022-02985-w

**Published:** 2022-04-04

**Authors:** Sung Eun Choi, Emily Mo, Nathan Palmer, Kathe Fox, John D. Da Silva, Shigemi Nagai, Jane R. Barrow

**Affiliations:** 1 Department of Oral Health Policy and Epidemiology, Harvard School of Dental Medicine, 188 Longwood Avenue REB 204, Boston, MA 02115 USA; 2 Department of Biomedical Informatics, Harvard Medical School, Boston, MA USA; 3 Department of Restorative Dentistry and Biomaterials Science, Harvard School of Dental Medicine, Boston, MA USA; 4 Department of Oral Medicine, Infection and Immunity, Harvard School of Dental Medicine, Boston, MA USA; 5 Office of Global and Community Health, Harvard School of Dental Medicine, Boston, MA USA

**Keywords:** Cognitive impairment, Edentulism, Dentures, Alzheimer's disease, Oral health

## Abstract

**Background:**

The scientific link between mastication strength and cognitive function has not yet been strongly corroborated in population studies. Utilizing large-scale claims, we aim to investigate the association between edentulism and cognitive impairment in older American adults.

**Methods:**

Using de-identified claims from a commercial insurer from 2015–2019, we conducted a retrospective cohort study using multilevel regression models to evaluate the association between denture status and clinically diagnosed cognitive impairment. Secondary analysis included symptomatic cognitive impairment in the outcome.

**Results:**

Adjusting for individual-level risk factors, denture status was significantly associated with clinical cognitive impairment with odds ratios of 1.13 (95%CI: 1.02–1.25) and 1.26, (95%CI: 1.09–1.45) for complete dentures on one or both jaws, respectively. Including symptomatic cognitive impairment in the analysis did not substantially change our fundamental findings.

**Conclusion:**

Prevention and treatment of oral diseases should be considered a key component in preserving the overall wellness of older adults.

**Supplementary Information:**

The online version contains supplementary material available at 10.1186/s12877-022-02985-w.

## Introduction

More than 16 million people in the US are living with cognitive impairment [1], and approximately 5.8 million adults aged 65 years or older in the US currently have Alzheimer’s disease [2], the most well-known form of cognitive impairment. With increased life expectancy, this number may rise to 13 million by 2050 [1]. Death attributable to Alzheimer’s disease increased by 67% from 2000 to 2013, becoming the sixth leading cause of death in the US [2]. While prevention of cognitive impairment has focused on controlling modifiable cardiovascular, dietary, and lifestyle factors [3], a growing body of literature suggests that oral conditions such as periodontal disease [4, 5] or simply general dental symptoms [6–8] are also associated with an increased risk of cognitive impairment.

There has been an increase in research evidence suggesting associations between tooth loss and cognitive impairment among older adults. It has been speculated that lowered mastication strength when chewing without natural teeth leads to a decrease in hippocampal stimulation, which can degrade cognitive health [9–11]. Observational studies on the association between tooth loss and cognitive decline have also been conducted, many of which point to lower cognitive function among people with more severe tooth loss [7, 12–14]. These studies mostly involve the observation of human subjects in very limited populations such as assisted living communities or small geographic regions worldwide, and may not be generalizable to larger populations or other locations, which has identified a need for more studies that can leverage larger-scale data to investigate this association. Existing studies on this association that utilized national claims data were only generalizable to their respective countries [15–17] and may not have been able to adjust for a wide range of potential confounders [17]. There are also studies that negate or are inconsistent with a positive link between tooth loss and cognitive impairment, [18–20] but such studies had limitations such as small sample size, potential selection bias, overadjustment bias, or lack of ability to generalize results beyond small communities. Therefore, evidence of such an association is inconclusive. In this study, we investigate the relationship between edentulism and risk of cognitive impairment across the US population, adjusting for potentially confounding medical conditions, using large-scale medical and dental claims data from a single US commercial health insurance provider.

## Methods

### Data source and study population

A retrospective cohort study was conducted using de-identified claims data from a single U.S. national insurer under managed care plan for the period January 1, 2015, through December 31, 2019. Our study population consisted of members who were 65 or older in 2015 and were enrolled for the entire period of 2015–2019 with at least one dental claim during the study period. Individuals who had either a complete denture on a single jaw, a complete denture on both jaws, or no denture-related claims at all with only partial dentures were excluded, resulting in 156,450 individuals aged between 65 and 105. Mean age of the study population was 72 $\pm 6.9$. This study was reviewed by the institutional review board of the Harvard Medical School and determined to be “not-human subjects research”. Methods followed the Strengthening the Reporting of Observational Studies in Epidemiology (STROBE) reporting guideline [21].

### Measures

Our primary outcome was a formal diagnosis of cognitive impairment, defined by a set of International Classification of Diseases (ICD) codes assigned for corresponding claims records. The list of ICD-9/10 codes used to define this clinical outcome includes dementia, Alzheimer’s disease, mild cognitive impairment, and corticobasal degeneration (list is available in Supplementary Table S1.) For those with edentulism, cognitive impairment diagnosed following the records of denture status was considered. A secondary outcome included only signs and symptoms of cognitive impairment, such as age-related cognitive decline and borderline mental functioning, without formal clinical diagnoses of cognitive impairment (ICD-9/10 codes listed in Supplementary Table S1).

Edentulism status was identified using Current Dental Terminology (CDT) codes associated with dental procedures (Supplementary Table S2). Three categories for edentulism were defined: 1) complete denture on both jaws, 2) complete denture on one jaw, or 3) natural teeth on both jaws. Individuals were sorted to denture categories corresponding to the most severe denture-related procedure codes that were inferable from the data during the study period. The group with complete dentures on one jaw included those with complete dentures on one jaw and natural teeth on the opposite side as well as those with complete dentures on one jaw and partial dentures on the opposite side. If an individual did not have any denture-related CDT codes in their claims data during the study period, they were assumed to have retained their natural teeth on both jaws.

To account for variation in the outcome variable and adjust for confounding, we adjusted for individual-level risk factors that are known to be associated with cognitive impairment: [3, 22–26] age at the beginning of the study period, diabetes, hypertension, high cholesterol, clinical depression, and history of smoking [27]. All of these conditions were identified with ICD-9/10 codes except for smoking. A combination of ICD-9/10 and/or Current Procedural Terminology (CPT) codes were used to identify smoking status (details in Supplementary Table S3). Due to lack of demographic information in the claims data aside from the patient zip code of residence, the zip code was used as a proxy that encompasses many if not all of the socioeconomic aspects of an individual [28], and has been shown to be an adequate proxy for individual level socioeconomic status. For individuals who had multiple zip codes listed in their enrollment information, we only counted the zip code that occurred the most frequently across their medical claims during the study period.

### Statistical analysis

Descriptive statistics of the characteristics and frequencies of cognitive impairment outcomes were reported by edentulism and risk factor status (Table 1). Unadjusted odds ratios (OR) for the clinically diagnosed cognitive impairment outcome were reported for each of the denture statuses and risk factors. To estimate the relationship between edentulism and clinically diagnosed cognitive impairment, controlling for potential confounding, we used a Bayesian multilevel logistic regression model, which is often used to aid in model convergence without needing to provide domain-specific prior information [29], to account for the grouping effects by geographic location (zip code) (Supplementary Text S1) using an implementation of the No-U-Turn sampling method (NUTS) in R (v. 3.6.1, R Foundation for Statistical Computing, Vienna, Austria). Adjusted ORs to account for potential confounding covariates listed in Sect. 1.2 were obtained and reported with 95% credible intervals for each covariate. Convergence of all coefficient estimates was confirmed using posterior trace and density plots (Supplementary Figures S1, S2, S4, S5). Multicollinearity among covariates was evaluated by assessing bivariate posterior scatterplots (Supplementary Figure S3, Supplementary Figure S6), which did not show a strong correlation between any pair of covariates.

**Table 1:** Population characteristics

Characteristic	Total population (%)	Any complete dentures (%)	Complete denture on both jaws (%)	Complete denture on one jaw (%)	Natural teeth (%)
All beneficiaries	156,450 (100)	7,933 (100)	2,210 (100)	5,723 (100)	148,517 (100)
No cognitive impairment	141,499 (90.44)	6,663 (83.99)	1,852 (83.30)	4,811 (84.06)	134,836 (90.79)
Clinical cognitive impairment	9,456 (6.04)	821 (10.35)	231 (10.45)	590 (10.31)	8,635 (5.81)
Clinical or symptomatic cognitive impairment	14,951 (9.56)	1,270 (16.01)	358 (16.20)	912 (15.94)	13,681 (9.21)

As a secondary analysis, we created an inclusive outcome of cognitive impairment by including clinical and symptomatic cognitive impairment statuses: The most severe category consisted of all individuals who had a clinical diagnosis of cognitive impairment regardless of whether they also had symptoms of cognitive impairment, the second most severe category consisted of individuals who only had signs and symptoms of cognitive impairment, and the third category consisted of those with no history of cognitive impairment. Unadjusted ORs were reported for this ordinal outcome using proportional odds models. Finally, a multivariate proportional odds model was fitted with this ordinal variable as the outcome, adjusting for the same covariates and using the same random effect structure as the primary analysis, and adjusted ORs were reported. Under the proportional odds assumption, the model estimated two equivalent odds ratios: 1) the odds of having clinical cognitive impairment as opposed to not having clinical cognitive impairment, and 2) the odds of having any clinical or symptomatic cognitive impairment as opposed to not having cognitive impairment.

## Results

### Descriptive statistics

Out of the 156,450 adults aged 65 or older included in this study, 6.04% were clinically diagnosed with cognitive impairment (as defined by our primary outcome) and 5.07% had a complete denture at some point during the study period. Among individuals with any complete dentures, the prevalence of clinical cognitive impairment was 10.35% as compared to 5.81% among those without dentures. Within the different denture groups, the prevalence of clinical cognitive impairment was 10.31% for those who had a complete denture on one jaw, and 10.45% for those who had a complete denture on both jaws (Table 1). When signs and symptoms of cognitive impairment were included in the outcome, the prevalence of clinical or symptomatic cognitive impairment was 16.01% among those with a complete denture on any jaw as compared to 9.21% among the group with no dentures. Within the two denture groups, the rate of clinical or symptomatic cognitive impairment was 15.94% for those who had a complete denture on one jaw, and 16.20% for those who had a complete denture on both jaws (Table 1).

### Unadjusted odds of cognitive impairment

Unadjusted association between clinical cognitive impairment and any complete dentures was significant with an OR of 1.89 [95% credible interval (CI): 1.75–2.04], compared to the natural teeth group (Table 2). The unadjusted OR of clinical cognitive impairment for those with a complete denture on both jaws was estimated to be 1.90 (95% CI: 1.64–2.19), and the unadjusted OR of clinical cognitive impairment for those with a complete denture on one jaw was estimated to be 1.88 (95% CI: 1.72–2.05). When regressing each unadjusted covariate on the ordinal cognitive impairment outcome, the OR of a higher severity of cognitive impairment for those with a complete denture on both jaws was estimated to be 1.92 (95% CI: 1.70–2.15), and the OR of a higher severity of cognitive impairment for those with a complete denture on one jaw was estimated to be 1.88 (95% credible interval: 1.74–2.02) (Table 2).

**Table 2:** Unadjusted odds ratios of risk factors for different cognitive impairment outcomes

		**Clinically diagnosed cognitive impairment **	Ordinal cognitive impairment
**Variable**	**% of cases in the study sample**	**Odds ratio (95% CI)**	**Odds ratio (95% CI)**
Age group of beneficiaries:			
65–69	47.89	Reference	Reference
70–79	36.40	4.43 (4.13–4.76)	3.28 (3.12–3.46)
80–89	13.04	18.43 (17.14–19.75)	12.23 (11.61–12.88)
90–99	2.61	38.14 (34.87–41.81)	26.30 (24.44–28.35)
100–109	0.06	34.87 (21.17–55.44)	29.17 (18.62–44.45)
Denture status of beneficiaries:			
Complete denture on both jaws	1.41	1.90 (1.64–2.19)	1.92 (1.70–2.15)
Complete denture on one jaw	3.66	1.88 (1.72–2.05)	1.88 (1.74–2.02)
Existing condition in beneficiaries:			
Diabetes	24.63	1.85 (1.77–1.93)	2.11 (2.04–2.20)
Hypertension	62.41	5.33 (4.98–5.70)	5.71 (5.41–6.02)
High Cholesterol	61.52	2.86 (2.71–3.03)	3.14 (3.01–3.27)
Depression	4.76	6.20 (5.86–6.56)	5.92 (5.62–6.24)
History of Smoking	17.26	1.69 (1.61–1.77)	1.96 (1.89–2.03)

Each of the risk factors were estimated to be significantly positively associated with cognitive impairment when unadjusted (Table 2), regardless of the cognitive impairment outcome. Out of all the risk factors considered excluding age groups, clinical depression and hypertension had the highest unadjusted ORs of cognitive impairment, which were respectively 6.20 (95% CI: 5.86–6.56) and 5.33 (95% CI: 4.98–5.70) for the clinical outcome, and 5.92 (95% CI: 5.62–6.24) and 5.71 (5.41–6.02) for the ordinal outcome.

### Multivariate analysis of clinically diagnosed cognitive impairment

When adjusting for all considered risk factors in our multivariate logistic regression model on clinically diagnosed cognitive impairment, we found strong evidence that the risk of cognitive impairment was higher for older adults with complete dentures, compared to the natural teeth group (Table 3). For those with complete dentures on both jaws, the OR was estimated to be 1.26 (95% CI: 1.09–1.45), and for those with complete dentures on only one jaw, the OR was estimated to be 1.13 (95% CI: 1.02–1.25). For each one-year increase in age, the odds of cognitive impairment were expected to increase by a factor of 1.15 (95% CI: 1.14–1.15). Most of the adjusted clinical risk factors had ORs and 95% credible intervals contained between 1 and 2, except for depression with an OR of 5.17 (95% CI: 4.84–5.50) and hypertension with an OR of 2.26 (95% CI: 2.10–2.44).

**Table 3:** Multilevel multivariate regression analysis: Association of risk factors with clinical cognitive impairment using a zip code-level random intercept model

Variable	Clinical cognitive impairment odds ratio (95% CI)	Cognitive impairment proportional odds ratio (95% CI)
	**Fixed effects**	
Denture status of beneficiaries:		
Complete denture on both jaws	1.26 (1.09–1.45)	1.26 (1.11–1.44)
Complete denture on one jaw	1.13 (1.02–1.25)	1.14 (1.05–1.24)
Risk factors:		
Age	1.15 (1.14–1.15)	1.13 (1.13–1.13)
Diabetes	1.24 (1.19–1.31)	1.39 (1.34–1.45)
Hypertension	2.26 (2.10–2.44)	2.51 (2.37–2.68)
High Cholesterol	1.43 (1.34–1.52)	1.45 (1.37–1.52)
Depression	5.17 (4.84–5.50)	4.83 (4.56–5.12)
History of Smoking	1.22 (1.15–1.29)	1.39 (1.33–1.45)
	**Ordinal intercepts**	
Baseline probability of clinical or symptomatic cognitive impairment		0.02 (0.01–0.02)
Baseline probability of clinical cognitive impairment		0.01 (0.01–0.01)
	**Random intercepts**	
Zip Code	Variance: 0.24	Variance: 0.23

### Multivariate analysis of ordinal cognitive impairment outcome

Our ordinal regression model assessed the proportional odds ratios for each denture status across binarizations of our categorical outcome of clinical, symptomatic, or no cognitive impairment (Table 3). For a beneficiary at the median age of 70 who has all their natural teeth and none of the risk factors, the probability of clinical or symptomatic cognitive impairment was estimated to be 0.02 (95% CI: 0.01–0.02), whereas the probability of only clinically diagnosed cognitive impairment was estimated to be 0.01 (95% CI: 0.01–0.01). For those with complete dentures on both jaws, the OR of being in a more severe category of cognitive impairment was expected to be 1.26 (95% CI: 1.11–1.44). For those with complete dentures on one jaw, the OR of being in a more severe category of cognitive impairment was estimated to be 1.14 (95% CI: 1.05–1.24). The ORs and 95% credible intervals for the risk factors remained between 1 and 2, with only the ORs for depression and hypertension exceeding 2.

## Discussion

Our analysis suggests that older American adults missing all of their natural teeth on at least one jaw are at significantly higher risk of a clinically diagnosed form of cognitive impairment, and that those who are missing all natural teeth are at even higher risk than those with a complete denture on only one jaw, accounting for age and common clinical risk factors, such as depression and hypertension. Our ordinal analysis suggests not only that the odds of having clinically diagnosed cognitive impairment are higher in older adults missing at least one full jaw of teeth, but also that the odds of having any symptoms or diagnosis of cognitive impairment are higher in older adults missing at least one jaw of teeth, accounting for age and common clinical risk factors.

A possible explanation for the mechanism relating edentulism to cognitive impairment is that cranial nerves bypass the blood–brain barrier, which is possible through the trigeminal nerve that innervates the masseter muscles. The masseter muscles are responsible for chewing and forming food boluses for swallowing with masticatory movement generated by the neuronal network in the brainstem [30]. It has been hypothesized that brain housekeeping molecules may travel from the masticatory muscles to the brain through the trigeminal nerve, the largest cranial nerve that exits the cranium carrying motor innervation to the muscles of mastication and receiving sensory inputs from proprioceptors of the teeth, after stimulation from mastication [17]. Clearance of amyloid-β reduces toxicity and results in maintenance of neuronal health, thereby, possibly preventing cognitive impairment.

Our study has an advantage over existing localized cohort studies on the same association [7, 8, 12–14, 18–20] due to its large sample size encompassing beneficiaries nationwide, which allowed us to reduce response bias and selection bias and adjust for more potential confounders without severely inflating uncertainty. By measuring cognitive impairment through claims data rather than cognitive tests such as the Mini-Mental State Examination (MMSE) [4, 6–8, 12–14, 19, 20], our study captured an image of patients’ cognitive state over the course of several years as opposed to a single snapshot in time, and we were also able to distinguish between patients who had received a formal diagnosis of cognitive impairment versus those who had only experienced symptoms. Additionally, our use of claims data to identify denture status may be less erroneous than self-reported measures of denture status and tooth loss [13, 19], which may be inaccurately reported by patients, especially those who are experiencing cognitive impairment. By identifying procedure codes for complete dentures, we were able to evaluate the increased risk of cognitive impairment for patients who had at least one complete jaw of missing teeth, with respect to patients who presumably retained all of their natural teeth. It is possible that some patients in the natural teeth group were actually missing a full jaw of natural teeth but did not have any denture-related claims due to affordability barriers, unenrollment, or other reasons that are not detectable based on the claims data—however, we would expect this to bias our results towards the null, and our results show a strong difference in cognitive impairment odds between the groups in spite of this potential bias. Compared to a former population-wide study based on Korean national health insurance claims [15], which ran separate analyses grouped by age and sex, our study summarizes a more general nationwide trend in the US due to our pooled analysis that adjusted for age. Additionally, our study examines the effects of high levels of edentulism, rather than simply examining the effect of any edentulism at all. Another study used Taiwanese national health insurance claims to assess the association between dementia and number of tooth extractions over a 17-year period [16]. By using denture status instead of tooth extraction count, our study accounts for total tooth loss and thus provides a better representation of mastication strength rather than only quantifying a patient’s most recent tooth loss history. It is important to relate edentulism severity to mastication strength because scientific findings have suggested that decreased mastication strength facilitates the development of dementia [9–11]. Our study supports this finding by identifying an association between reduced mastication and reduced cognitive function within the US population. A recent study based on denture treatment codes in Japanese national healthcare data found higher unadjusted odds ratios of cognitive impairment for patients with weaker mastication levels than what we estimated after adjusting for potential confounding factors [17]. While this study analyzed the mastication at a granular level by utilizing electronic health records, it fails to adjust for additional confounding factors and grouping effects by geographic location.

Our study has some limitations. First, we used administrative claims data with varying completeness of documentation, such as incomplete information on dental insurance beneficiary status. By including all individuals who have ever received any dental services in the analysis, we could be missing those with dental coverage who never used any dental services or including those who only used dental services for part of the study period; thus, our study could potentially underestimate the oral health conditions of individuals. Similarly, cognitive impairment may be underdiagnosed in our cohort attributable to the nature of administrative claims data; claims data is a reliable record of the care received by the beneficiaries but does not provide information on the care needed [31]. Like any observational studies using administrative claims data, our results may not be generalizable beyond the study population and only represent statistical significance of the association between edentulism and cognitive impairment. Despite the odds ratios and credible intervals being significantly positive, our study does not assure that the results are clinically relevant. Only under the assumption of missing at random would our study not create a bias. Second, due to lack of patient chart information in the claims data, we used denture status as a proxy for number of teeth missing, rather than being able to evaluate missing teeth as a continuous exposure variable. Although this approach has been used in prior studies [15], dental diagnostic information from electronic health record data would allow us to perform a more refined study. Third, because the US dental claims do not contain diagnostic codes, but only procedures, denture status was indirectly inferred in our study. If a patient lacked complete denture CDT codes, it was difficult to determine the highest extent of their tooth loss over the study period, since partial denture CDT codes and miscellaneous CDT codes represent a very wide spectrum of tooth loss, so we excluded patients who had record of only partial dentures or other procedures that did not correspond to complete dentures. In excluding partial denture wearers, however, we were unable to evaluate whether lower levels of mastication impact the prevalence of cognitive impairment. We also may have excluded many people who were almost as edentulous as those included in the study, but who simply lacked a complete denture. Fourth, prior studies reported a positive relationship between edentulism and cognitive impairment adjusting for behavioral and socioeconomic factors [12, 13], such as physical exercise, race/ethnicity, income, and education; however, due to lack of information on these variables in the claims data, they could not be directly included in the analysis, and we were only able to roughly account for socioeconomic status by using zip code as a random effect [28, 32]. Without detailed patient characteristics and shared risk factors between cognitive impartment and edentulism incorporated in the analysis, it remains unclear whether the observed association between denture status and cognitive impairment represents a causal relationship or is due to the confounding effects of other variables such as low socio‐economic status. Direction of causality is also not inferable from our results. Large randomized intervention trials or observation studies that minimize potential bias of confounding are needed to establish a robust causal relationship. Lastly, while our study population covers a large and geographically diverse group of beneficiaries, it is limited to a single insurance provider and may not be generalizable to the general US older adult population.

Our findings suggest a higher likelihood of cognitive impairment among older Americans who were missing teeth on one or both jaws compared to those who retained all of their natural teeth. While more controlled research is needed to demonstrate this association as causal on a national scale, our findings agree with existing literature that decreased mastication with natural teeth causes reduced hippocampal stimulation, which can lead to memory problems. Timely prevention and treatment of oral diseases should be considered as a key component in preserving the overall wellness of older adults.

## Supplementary Information

**Additional file 1.**

## Data Availability

Data user agreements prohibit the authors from making the dataset publicly available. Access may be granted to those who meet pre-specified criteria for confidential access upon mutual agreements with data provider. The analytic codes are available from the authors upon request.
