# Supplementary material for: Cognitive impairment and edentulism among older adults: an observational study using claims data
Source: BMC Geriatr. 2022 Apr 4;22:278. doi: 10.1186/s12877-022-02985-w (PMC8981850; doi:10.1186/s12877-022-02985-w)

**Supplemental Material**

Supplementary Table S1. Definition of cognitive impairment outcomes

Supplementary Table S2. Definition of denture status

Supplementary Table S3. Definition of risk factors status

Supplementary Text S1. Bayesian modeling

Supplementary Figure S1. Density plot for logistic regression model

Supplementary Figure S2. Trace plot for logistic regression model

Supplementary Figure S3. Bivariate posterior scatterplots for logistic regression model

Supplementary Figure S4. Density plot for ordinal regression model

Supplementary Figure S5. Trace plot for ordinal regression model

Supplementary Figure S6. Bivariate posterior scatterplots for ordinal regression model

**Supplementary Table S1. Definition of cognitive impairment outcomes**

If a beneficiary had any ICD-9/10 codes in their claims data during the study period listed in the following table, they were classified as having the corresponding cognitive impairment outcomes. An “X” at the end of an ICD code indicates that all ICD codes starting with the preceding characters were included.

| **Cognitive Impairment Outcome** | **ICD-10** | **ICD-9** |
| --- | --- | --- |
| Clinical Outcome | F03, G30, G31.0, G31.83, G31.84, G31.85 | 294.20, 331.0, 331.19, 331.6, 331.82, 331.83 |
| Symptomatic Outcome | R41.81, R41.82, R41.83, R41.84X, R41.89 | 797, 780.97, 799.5X |

**Supplementary Table S2. Definition of denture status**

The following table defines the logic used to assign each beneficiary to a denture category based on their history of dental claims during the study period. Note that CDT codes listed in the table only include those that appeared in our claims data.

| **Denture Status** | **CDT Code Classification Rule** |
| --- | --- |
| Complete denture on both jaws | Has a claim from **each** of the following groups:  (D5110, D5130, D5410, D5512, D5710, D5730, D5750, D5810, D5863)  AND  (D5120, D5140, D5411, D5511, D5711, D5731, D5751, D5811, D5865) |
| Complete denture on one jaw | Ruled out of the “complete denture on both jaws” group, AND  Has a claim from the following group: (D5110, D5120, D5130, D5140, D5410, D5411, D5510, D5511, D5512, D5520, D5710, D5711, D5730, D5731, D5750, D5751, D5810, D5811, D5860, D5863, D5865, D5876) |
| No denture (natural teeth) | Has no CDT codes from D5*** category |

**Supplementary Table S3. Definition of risk factors status**

If a beneficiary had any ICD-9/10 codes in their claims data during the study period listed in the following table, they were classified as having the corresponding risk factors. An “X” at the end of an ICD code indicates that all ICD codes starting with the preceding characters were included.

| **Risk Factor** | **ICD-10** | **ICD-9** | **CPT** |
| --- | --- | --- | --- |
| Diabetes | E08.X, E09.X, E10.X, E11.X, E12.X, E13X | 250.X |  |
| Hypertension | I10.X | 401.X |  |
| High Cholesterol | E78.X | 272.0 |  |
| Depression | F33.X | 296.3 |  |
| History of smoking | Z87.891, Z72.0, F17.200 | V15.82, 305.1 | G0375, G0376, 99406, 99407 |

**Supplementary Text S1. Bayesian modeling**

Our Bayesian analyses were performed using the rstanarm and brms packages for R, which both rely on Stan. A Bayesian method was chosen because of its improved convergence compared to the maximum likelihood method. Weakly informative prior distributions for all of the coefficients were specified as Normal(0, 1) which symbolizes neutral prior beliefs about the covariates’ relationship with cognitive impairment while simultaneously avoiding extreme odds ratio estimates. Priors for model intercepts were specified as Normal(0, 2.5) when the covariates are centered at their means. Priors for the random effect variance were specified as Gamma(1, 1). The NUTS sampler ran on 4 separate Monte Carlo Markov chains, each with 1200 warmup iterations and 600 sampling iterations. For the primary analysis, since we had first fit a model with the same fixed and random effect structure using the maximum likelihood method which did not converge, we offset each of the fixed effect coefficient estimates from this model with different Normal(0, 1) distributed errors, and set these estimates as the starting point for each chain. After the models were fit, we confirmed that the parameter estimates were stable by assessing the posterior density plots (Supplementary Figure S1 and S4), sampler trace plots (Supplementary Figure S2 and S5), and autocorrelations for each parameter. The patterns in the trace plots all appeared to be random and unassociated with previous values, and the density plots appeared roughly Normally distributed, implying that the parameter estimates had converged successfully. Finally, we assessed bivariate posterior scatterplots for each pair of fixed effects (Supplementary Figure S3 and S6) in order to check for multicollinearity.

**Supplementary Figure S1. Density plot for logistic regression model**


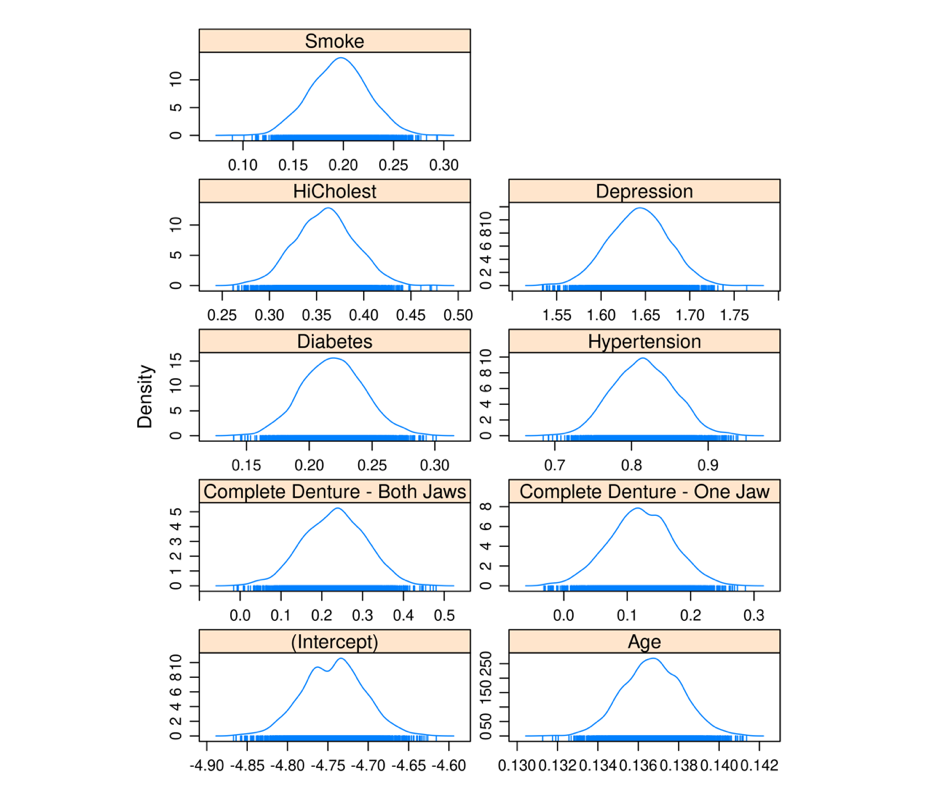


**Supplementary Figure S2. Trace plot for logistic regression model**


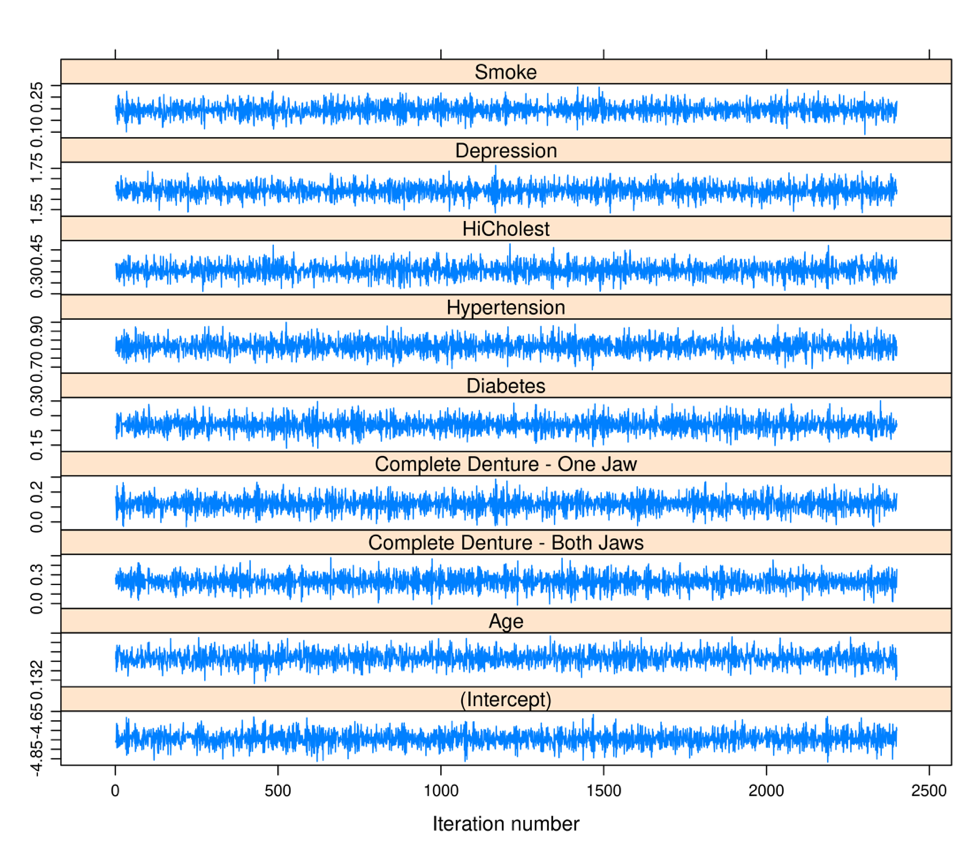


**Supplementary Figure S3. Bivariate posterior scatterplots for logistic regression model**


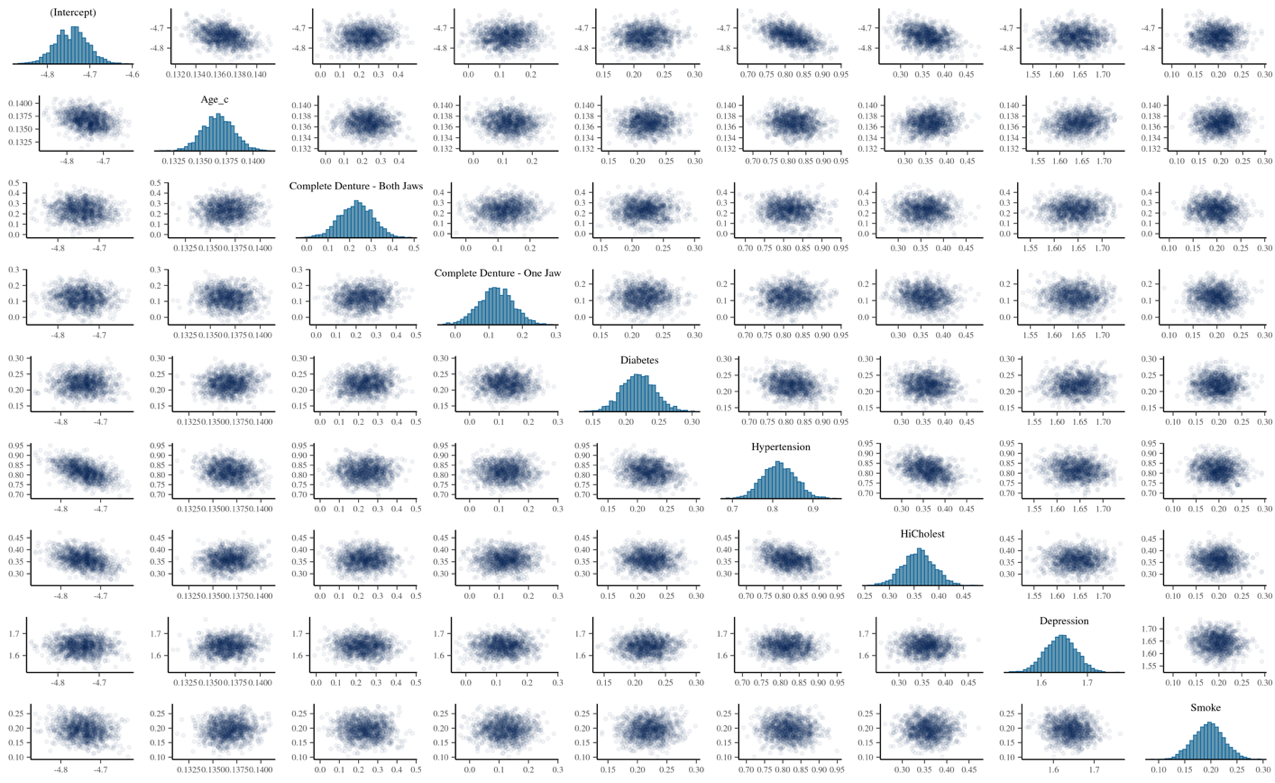


**Supplementary Figure S4. Density plot for ordinal regression model**


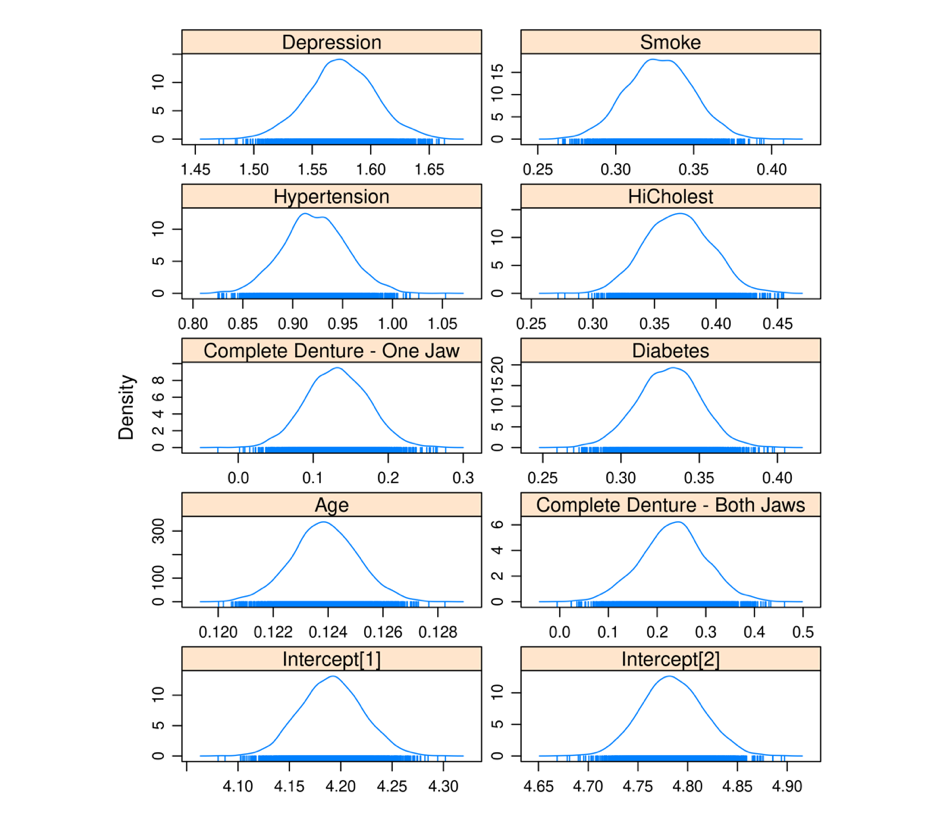


**Supplementary Figure S5. Trace plot for ordinal regression model**


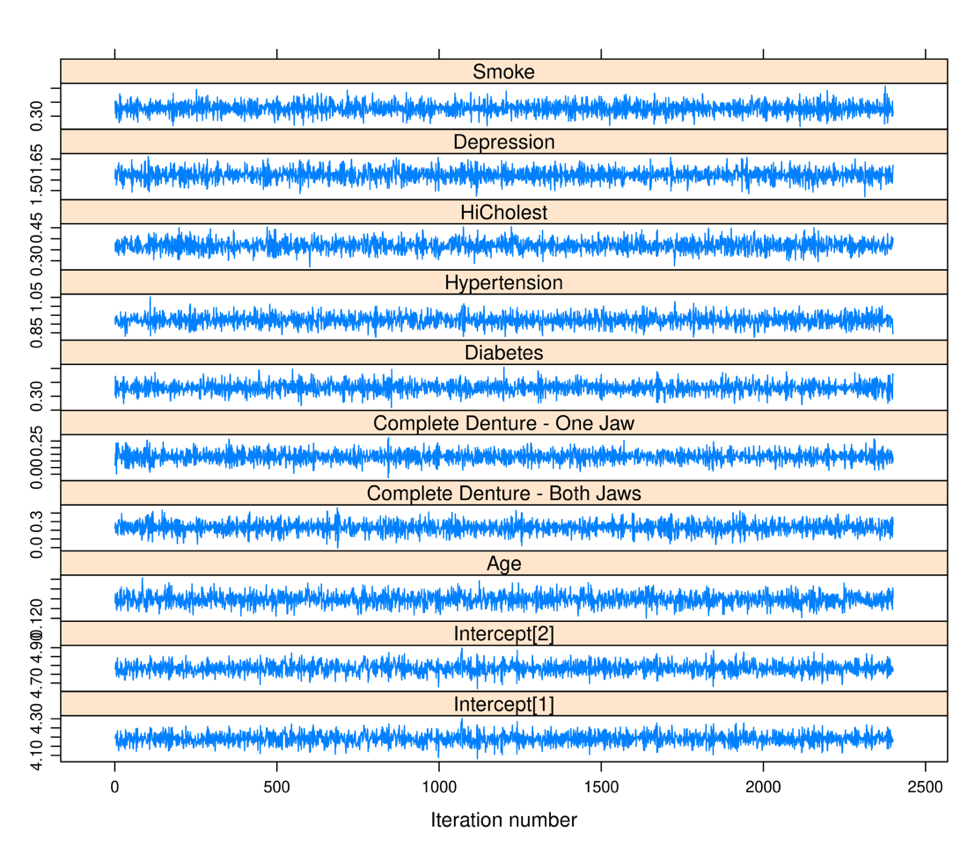


**Supplementary Figure S6. Bivariate posterior scatterplots for ordinal regression model**


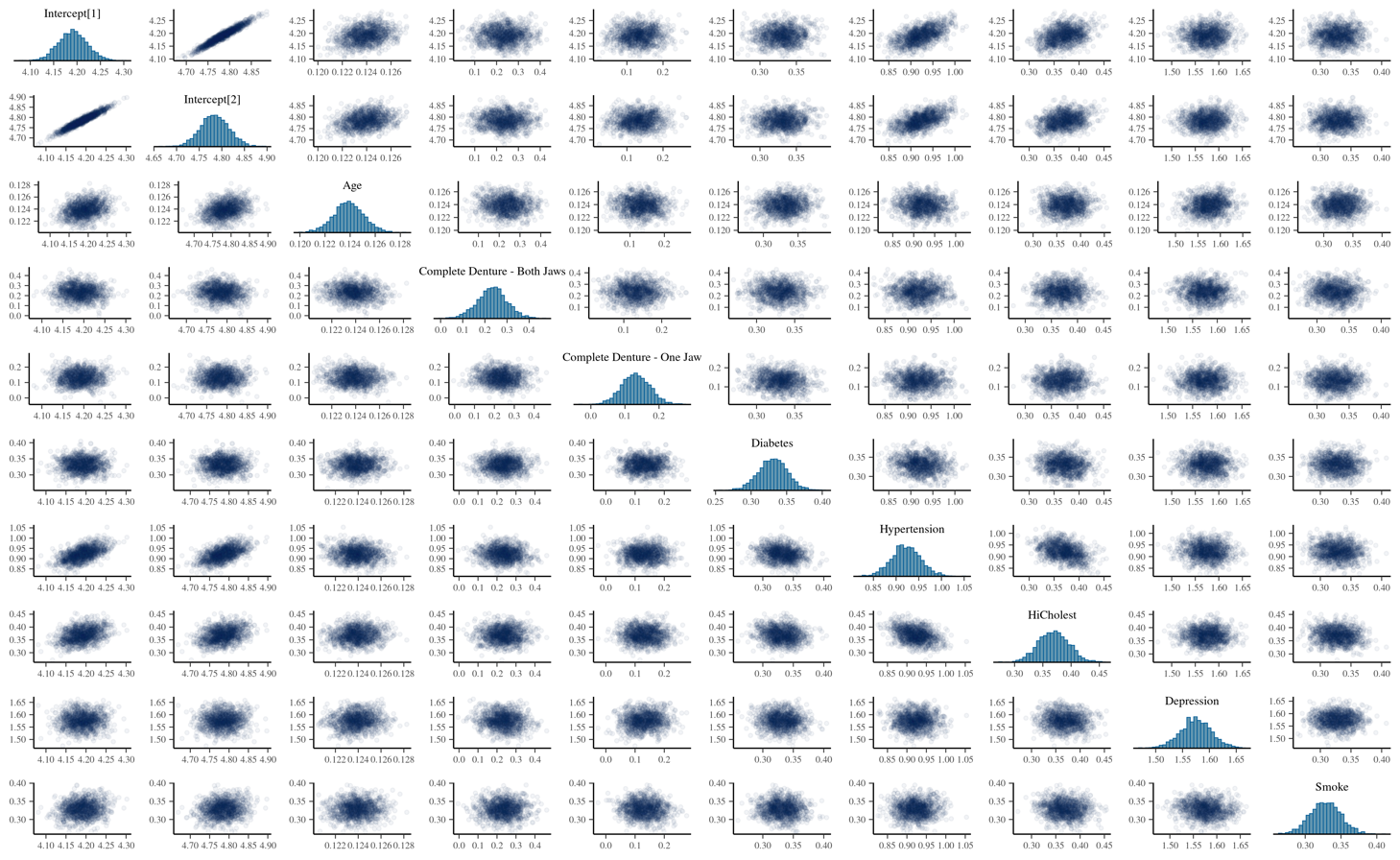

Supplement: Supplementary file 1 — Additional file 1. [file 12877_2022_2985_MOESM1_ESM.docx]
